# An Unbiased Machine Learning Exploration Reveals Gene Sets Predictive of Allograft Tolerance After Kidney Transplantation

**DOI:** 10.3389/fimmu.2021.695806

**Published:** 2021-07-08

**Authors:** Qiang Fu, Divyansh Agarwal, Kevin Deng, Rudy Matheson, Hongji Yang, Liang Wei, Qing Ran, Shaoping Deng, James F. Markmann

**Affiliations:** ^1^ Organ Transplantation Center, Sichuan Provincial People’s Hospital and School of Medicine, University of Electronic Science and Technology of China, Chengdu, China; ^2^ Center for Transplantation Sciences, Massachusetts General Hospital, Harvard Medical School, Boston, MA, United States; ^3^ Division of Transplantation, Department of Surgery, Hospital of the University of Pennsylvania, Philadelphia, PA, United States

**Keywords:** machine learning, kidney transplantation, PBMC, tolerance, biomarker

## Abstract

Efforts at finding potential biomarkers of tolerance after kidney transplantation have been hindered by limited sample size, as well as the complicated mechanisms underlying tolerance and the potential risk of rejection after immunosuppressant withdrawal. In this work, three different publicly available genome-wide expression data sets of peripheral blood lymphocyte (PBL) from 63 tolerant patients were used to compare 14 different machine learning models for their ability to predict spontaneous kidney graft tolerance. We found that the Best Subset Selection (BSS) regression approach was the most powerful with a sensitivity of 91.7% and a specificity of 93.8% in the test group, and a specificity of 86.1% and a sensitivity of 80% in the validation group. A feature set with five genes (HLA-DOA, TCL1A, EBF1, CD79B, and PNOC) was identified using the BSS model. EBF1 downregulation was also an independent factor predictive of graft rejection and graft loss. An AUC value of 84.4% was achieved using the two-gene signature (EBF1 and HLA-DOA) as an input to our classifier. Overall, our systematic machine learning exploration suggests novel biological targets that might affect tolerance to renal allografts, and provides clinical insights that can potentially guide patient selection for immunosuppressant withdrawal.

## Background

While modern immunosuppressive treatments have significantly improved the graft survival of kidney transplantation, they also result in a multitude of unwanted side effects including increased susceptibility to infection, chronic allograft injury, and malignancy (1, 2). Operative tolerance, a state of long-term allograft acceptance without continuous immunosuppression, is an important tenet for the success of solid organ transplantation (3, 4). Numerous studies on tolerance have been conducted to find biomarkers in peripheral blood mononuclear cells (PBMCs) predictive of renal allograft tolerance (3, 5–10). Spontaneous allograft tolerance in kidney transplantation appears to be far less frequent than in liver transplantation (3, 5, 11), limiting the numbers of patients available for tolerance biomarker studies. Although prior meta-analysis investigations have sought to identify key biomarkers for tolerance (3, 5–8, 12), the potential influence of the diverse RNA sequencing platforms used across the existing studies has remained a confounding variable. Several published methods address the problem of integrating data across platforms (13, 14). Nevertheless, when sequencing platforms vary, the sample management and gene expression profiles can differ substantially. For instance, the same probe may yield different gene segments on different platforms. This limitation can be addressed, however, by combining the different existing databases based on the same platform, which would maintain the probes and exactly match the genes mapped.

Recent advances in machine learning (ML) have allowed for efficient models for prediction, which can detect novel hidden patterns within large biomedical databases more effectively than conventional methods (15, 16). ML can be especially powerful when nonlinear interactions between the predictors exist in a high-dimensional feature space (15, 17). Indeed, ML is starting to become widely applicable for kidney disease prediction and identifying at-risk individuals in a variety of clinical scenarios (3, 10, 18). Nonetheless, the predictive power of most existing ML studies tends to vary from model to model, and any parameter changes in the algorithm can significantly impact the final prediction or model output. Although existing allograft tolerance studies have generated many biologically relevant gene lists, the overlap among these different studies is generally poor (12), likely due to small sample sizes, as well as inconsistent models and parameters.

Here, we present an ML-based analytical solution that circumvents many of the aforementioned challenges by combining existing genomic microarray databases based on the same platform (GPL570). Using PBMC samples from 63 tolerant patients (19 in the training group, 12 in the test group, 22 in Immune Tolerance Network (ITN), and 10 in Indices of Tolerance (IOT), we systematically compare 14 different prediction models to determine the optimal model parameters and key gene features that are consistently predictive of renal allograft tolerance. Altogether, our unbiased ML approach successfully mines for features that are robustly associated with renal allograft tolerance, and suggests the optimal timing of immunosuppressant withdrawal with a low risk of acute or chronic rejection.

## Materials and Methods

### Microarray Data Pre-Processing

Publicly available PBMC microarray data on tolerance studies after renal transplantation using the GPL570 platform (Affymetrix Human Genome U133 Plus 2.0 Array) were downloaded from the GEO database (www.ncbi.nlm.nih.gov/geo/). Tolerant (TOL) recipients were defined as patients who had not received immunosuppression, with stable renal function (serum creatinine levels < 25% of baseline or < 150 lmol/L) for at least 1 year. Stable function (STA) recipients were patients who took standard immunosuppression (SI) and had stable renal function (same criteria as TOL) for at least 1 year. Lastly, healthy volunteers (HV) were individuals with a normal white blood cell count, and no known history of renal/concomitant diseases for at least 6 months prior to the study. TOL and STA samples were based on a histopathologic examination more than 6 months post-transplantation. The ratio of STA and TOL samples included was approximately 1:1. The gene expression matrix was normalized using the gcRMA algorithm (19). After k-Nearest Neighbor (KNN) imputation (using the *R* package impute) (20) for the raw expression data matrix, surrogate variable analysis was applied to adjust for batch effects (21). Differentially expressed genes (DEGs) were analyzed using the Empirical Bayes method based on limma (22). Log2 absolute fold change >1 (FDR adjusted *P* < 0.05) was set as the cut-off value to identify significant DEGs. The Go enrichment analysis was performed in *R* (*version 3.6.3*) and the codes are available on GitHub (https://github.com/wangshisheng/EnrichVisBox). Finally, hierarchical clustering analysis was performed using one minus Pearson’s correlation coefficient method.

### Establishment of Predictive Models

DEGs identified were used to predict stable (STA) or tolerant (TOL) status. The dataset was divided into training and test sets by 3:2 randomization without replacement. Fourteen models were established to assess the predictive accuracy of tolerance: Logistic Regression (LR), Linear Discriminant Analysis (LDA) (23), Quadratic discriminant analysis (QDA), Multivariate Adaptive Regression Splines (MARS) (24), best subset selection (BSS, leaps package), ridge regression (25), elastic network (E-Net), the lasso regression (Lasso), kNN Classification (23), support vector machine (SVM) with radial basis function (RBF) kernel (package e1071), classification tree (package rpart), random forest (package randomForest) (26), and eXtreme Gradient Boosting (XGBoost) (27). The packages indicated in parentheses are available open source in *R version 3.6.3.* In addition to supervised ML methods, the unsupervised principal component analysis (PCA) was also utilized (28), as a measure of comparison for the performance of the ML methods being tested.

### Assessment of Prediction Models and Validation of Key Gene Features Predictive of Tolerance

Classification algorithms have to employ a balance penalizing poorly predictive variables and overfitting the data. To minimize any overfitting or underfitting, different methods to corroborate our observations were applied. For example, Bayes’ theorem was adopted for LDA model prediction. For the MARS model, k-fold cross-validation (k=3) was applied, and the additive model was repeated without interactions. Bayesian Information Criterion (BIC) was used to establish the most optimal BSS model. Leave-one-out cross-validation (LOOVE) was used as the resampling method, and α and λ combinations were exhausted by grid search (using the *R* package caret) and selected in the training group in the E-Net model. In Ridge, Lasso, and E-Net models, the k-fold cross-validation (k=5) was introduced using the glmnet package. LOOVE and caret were also used in KNN to select the optimal k. Kappa, calculated as (probability of agreement - probability of expected outcome)/[1 - (probability of expected outcome)], was used to evaluate the efficiency of a model. Furthermore, to enhance the accuracy of the different ML models, several weighted distance methods, including *rectangular*, *triangular*, and *epanechnikov* in weighted kNN (KKNN package) were examined in the KNN model. We also employed different kernels, including the linear kernel, polynomial kernel, RBF kernel, and sigmoid kernel, for the SVM model. The Gini index is a robust measure of the dispersion of a variable’s distribution, and Gini weighting has been shown to provide a sensitive method of feature selection, including with kernel ML algorithms (29). Thus, the Gini index was used to improve the efficiency of the classification tree. For the XGboost model, the k-fold cross-validation (k=5) and the caret package were utilized to resample data and tune parameters. ROC curve or the confusion matrix, including area under the curve (AUC), accuracy, sensitivity, specificity, positive predictive value (PPV), and negative predictive value (NPV) were calculated.

### Prediction Assessment and Validation of Renal Graft Rejection

The predictive power of the different models was validated in GSE14655 (data set 2, based on GPL8136), including 22 TOL samples collected by the ITN in the United States, and 10 TOL samples by the IOT in Europe. The cutoff point differs when sequencing platforms vary. We calculated the new optimal cutoff in ITN using the 5-gene signature, and validated the findings in IOT (7). The expression values of the 5-gene signature above the cutoff were classified as TOL, those below as STA. To assess the prediction of graft rejection, the expression data in GSE21374 (n=282) were transformed into Z scores (30). Z score > 1.0 was set as the cut-off for high expression, Z score < -1.0 was set as the cut-off for low expression, and Z scores between -1.0 and 1 were defined as normal or mean expression. Each gene was tested independently *via* Cox regression using the RegParallel package (https://github.com/kevinblighe/RegParallel) in *R*.

### Statistical Analysis

Kaplan-Meier and log-rank methods were used for graft rejection prediction. Shapiro-Wilk normality test or Kolmogoov-Smirnov test was used to assess whether the data belong to a Gaussian distribution. Differences between TOL and STA groups were analyzed using the Student’s *t*-test or Kolmogorov-Smirnov test. FDR-adjusted *P* < 0.05 was considered statistically significant. All data analysis was performed in the statistical programming language *R* (version 3.6.3).

## Results

### Identification and Analysis of Differentially Expressed Genes

**Figure 1:**
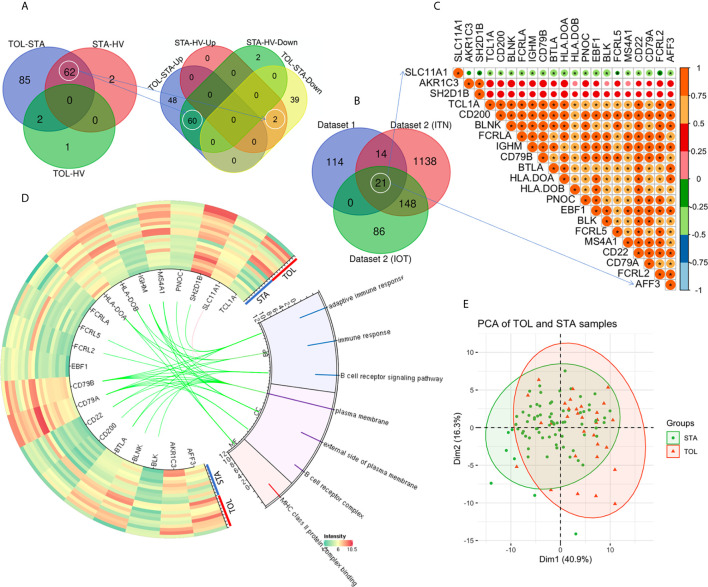
Identification of differentially expressed genes. **(A)** Venn diagram of the DEGs in dataset 1 is shown. **(B)** There were 21 co-DEGs among dataset 1, ITN and IOT in dataset 2. **(C)** Pearson correlation analysis of the 21 co-DEGs is shown. **(D)** The heatmap and Gene Ontology (GO) enrichment analysis of the co-DEGs in dataset 1 is demonstrated. **(E)** The top 2 PCs were used to display the characteristics of STA and TOL. *P < 0.05.

Due to the inherently low number of TOL subjects profiled in existing studies of renal allograft tolerance, we included fewer STA samples in the training dataset to maintain a 1:1 ratio of STA: TOL samples for designing our ML classifiers. PBMC gene expression data from 31 TOL samples, 39 STA samples, and 24 HV samples across GSE22707 (8) and GSE22229 (3) were combined in dataset 1. Data from 32 TOL samples (22 in ITN, and 10 in IOT), and 60 STA samples were used for validation. The demographic characteristics of the patient cohorts are shown in **Supplementary Table S1**. Compared to the STA samples, there were 149 DEGs (Log2|fold change| > 1 and adjusted *P* < 0.05) in the TOL samples, among which 108 transcripts were upregulated and 41 transcripts were downregulated (**Figure 1A**). Compared to the HV samples, there were 64 DEGs in the STA samples (2 upregulated and 62 downregulated transcripts), and 3 DEGs in the TOL samples (1 upregulated and 2 downregulated transcripts, **Figure 1A**). Interestingly, TUBB2A and TUBB2B were upregulated in STA PBMCs when compared with HV and TOL PBMCs. Additionally, 60 genes were found to be significantly downregulated in STA PBMCs when compared with HV and TOL PBMCs (**Figure 1B**). Two genes –EGR1 and EIF5/SNORA28– were downregulated in TOL PBMCs when compared with HV and STA PBMCs. There were 21 co-DEGs among dataset 1 and dataset 2 (ITN and IOT respectively, **Figure 1B**, and **Supplementary Figure S1**, **Table S2**). Gene Ontology (GO) analysis revealed that seven pathways were significantly enriched, five of which were B cell-related (**Figure 1C**). Pearson’s correlation analysis of the identified DEGs is shown in **Figure 1D**, and the characteristics of STA and TOL samples are displayed in **Figure 1E** using the top 2 principal components (PCs).

### Comparative Analysis of the Predictive Power of Linear Models

**Figure 2:**
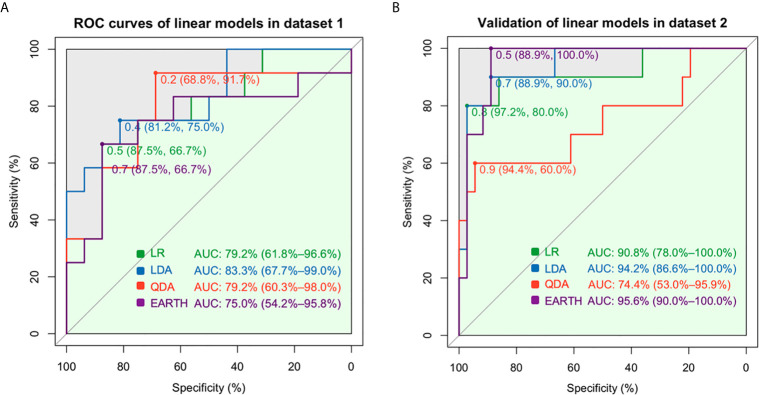
Prediction assessment and validation of the linear models. **(A)** The AUCs were 79.2% for the LR, 83.3% for LDA, 79.2% for QDA, and 75.0% for MARS in dataset 1. **(B)** The AUCs were 90.8% for LR, 94.2% for LDA, and 74.4% for QDA in dataset 2.

**Figure 3:**
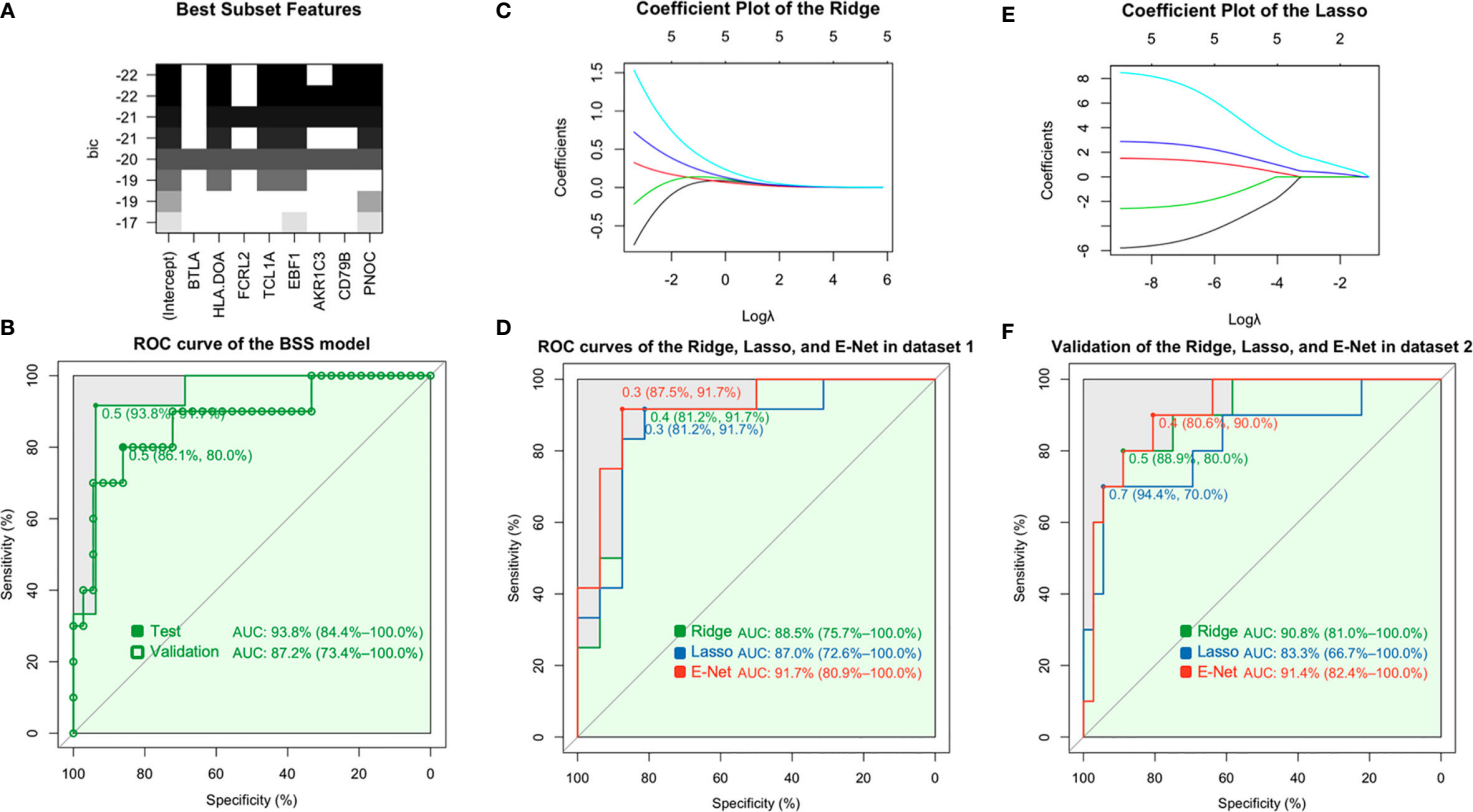
Prediction assessment and validation of BSS, Ridge, Lasso, and E-Net. **(A)** A minimum BIC score was obtained when five gene features (HLA-DOA, TCL1A, EBF1, CD79B, and PNOC) were included. **(B)** The AUC was 93.8% for BSS in dataset 1, and 87.2% in dataset 2. **(C)** The coefficient plot of the Ridge model is shown. **(D)** An AUC of 88.5% for the Ridge, 87.0% for the Lasso, and 91.7% for the E-Net was obtained in dataset 1. **(E)** The coefficient plot of the Lasso model is shown. **(F)** AUC values of 90.8% for Ridge, 83.3% for Lasso, and 91.4% for E-Net were obtained in dataset 2.

Among linear models, the AUC values were 79.2% for LR, 83.3% for LDA, 79.2% for QDA, and 75.0% for MARS in dataset 1 when seven gene features (BTLA, FCRL2, TCL1A, EBF1, AKR1C3, CD79B, PNOC) were included (**Figure 2A**). The generalizability and prediction of tolerance by these models were validated in dataset 2, wherein the AUC for LR was 90.8%, LDA was 94.2%, QDA was 74.4%, and MARS was 95.6% (**Figure 2B**). An AUC of 93.8% was obtained for BSS in dataset 1 and 87.2% in dataset 2 when five gene features (HLA-DOA, TCL1A, EBF1, CD79B, and PNOC, **Supplementary Table S2**) were included (**Figures 3A, B**), whereas an AUC of 88.5% was obtained for Ridge regression using same gene features (**Figures 3C, D**). In contrast, the Lasso model had an AUC of 87.0%, compared to an AUC of 91.7% with E-Net (**Figure 3E**). The AUC for Ridge was 90.8%, for Lasso was 83.3%, and for E-Net was 91.4% in dataset 2 (**Figure 3F**). The Ridge, Lasso, and E-Net models were then validated with a class/auc type measure. After k-fold cross-validation (k=5), the Ridge model attained the minimum binomial deviance using logλmin (minimum standard error, **Supplementary Figure S2A**). The maximum AUC was achieved using logλmin when the type measure was auc/class in dataset 1 (**Supplementary Figures S2B, C**). ROC analysis showed consistently high AUC values irrespective of whether the logλmin or logλ1se (one standard error away from the minimum standard error) was used in dataset 2 (**Supplementary Figure S2D**). The Lasso model resulted in minimum binomial deviance when logλmin was used and four genes were included (**Supplementary Figure 2E**). The maximum AUC was achieved with this four-feature gene set when type measure was “auc” in datasets 1 and 2 (**Supplementary Figures S2F–H**). Because Lasso uses an L1 regularization which shrinks noninformative feature coefficients to zero, it has inherently fewer gene features, which may lend itself to easier translational applications. Similarly, consistently high AUC values were obtained with the E-Net models using logλmin or logλ1se (**Supplementary Figures S2I–K**). The E-Net model with 5 genes obtained the minimum binomial deviance and the maximum AUC using logλmin and an auc type measure (**Supplementary Figures 2I–K**).

### Prediction Assessment and Validation of Nonlinear Models

Among the nonlinear models, kernel SVM had an accuracy of 85.7% and a Kappa value of 0.67 in dataset 1, and an 89.1% accuracy in dataset 2 (**Table 1**). In contrast, the random forest model obtained a minimum error and an accuracy rate of 71.43% (trees = 12; **Table 1** and **Supplementary Figure S3A**). The Gini index was used to weigh features as described in the methods, and gene features used in the classification tree were those with the highest MeanDecreaseGini values (**Supplementary Figure S3B**). Meanwhile, XGBoost had an AUC of 84.1% in dataset 1 and 92.2% in dataset 2 (**Supplementary Figure S3C**); the additive benefits of including more features in the model input are shown in **Supplementary Figure S3D**. Although the classification tree had a sensitivity and specificity > 80.0%, based on computational efficiency and overall accuracy, SVM with a linear kernel appeared to be the best model for tolerance prediction using five gene features.

**Table 1:** Confusion Matrix and Statistics using KNN, KKNN, SVM, Classification Tree, and Random Forest.

Model	Dataset	Prediction	Reference		Accuracy %	Kappa	Sensitivity %	Specificity %	PPV %	NPV %
			STA	TOL						
KNN	Dataset 1 Test	STA	11	3	71.43	0.43	75.00	68.75	64.29	78.57
		TOL	5	9						
	Dataset 2 Validation	STA	30	3	80.43	0.48	70.00	83.33	53.85	90.91
		TOL	6	7						
KKNN	Dataset 1 Test	STA	12	3	75.00	0.49	75.00	75.00	69.23	80.00
		TOL	4	9						
	Dataset 2 Validation	STA	30	3	80.43	0.48	70.00	83.33	53.85	90.91
		TOL	6	7						
SVM linear tune	Dataset 1 Test	STA	13	1	85.71	0.71	91.67	81.25	78.57	92.86
		TOL	3	11						
	Dataset 2 Vaidation	STA	34	3	89.13	0.67	70.00	94.44	77.78	91.89
		TOL	2	7						
SVM polynomial	Dataset 1 Test	STA	12	3	75.00	0.49	75.00	75.00	69.23	80.00
		TOL	4	9						
	Dataset 2 Validation	STA	33	4	84.78	0.54	60.00	91.67	66.67	89.19
		TOL	3	6						
SVM radial	Dataset 1 Test	STA	10	3	67.86	0.36	75.00	62.50	60.00	76.92
		TOL	6	9						
	Dataset 2 Validation	STA	33	4	84.78	0.54	60.00	91.67	66.67	89.19
		TOL	3	6						
SVM sigmoid	Dataset 1 Test	STA	15	4	82.14	0.62	66.67	93.75	88.89	78.95
		TOL	1	8						
	Dataset 2 Validation	STA	31	2	84.78	0.60	80.00	86.11	61.54	93.94
		TOL	5	8						
Classification Tree (party)	Dataset 1 Test	STA	14	3	82.14	0.63	75.00	87.50	**81.82**	**82.35**
		TOL	2	9						
	Dataset 2 Validation	STA	35	5	86.96	0.55	50.00	97.22	83.33	87.50
		TOL	1	5						
Random Forest	Dataset 1 Test	STA	12	4	71.43	0.42	66.67	75.00	66.67	75.00
		TOL	4	8						
	Dataset 2 Validation	STA	31	2	84.78	0.60	80.00	86.11	61.54	93.94
		TOL	5	8						

bold values: both PPV% and NPV% > 80%.

Next, we used PCA to compare this unsupervised method with the other supervised and semi-supervised ML methods used in our analysis. Using the same five genes – HLA-DOA, TCL1A, EBF1, CD79B, and PNOC – we found that the first three PCs accounted for ~80% variance in the data (**Supplementary Figure S4A**), and separated TOL from STA patients in the test groups (**Supplementary Figure S4B**). Using the top three PCs, the model achieved an AUC of 84.4%, sensitivity of 83.3% and a specificity of 87.5% (**Supplementary Figure S4C**). This model performed reasonably well in the validation dataset 2 with 90% sensitivity and a 77.8% specificity, accurately classifying the TOL and STA patients (**Supplementary Figures S4D, E**).

### Prediction of Graft Rejection and Cox Proportional Hazards Analysis

**Figure 4:**
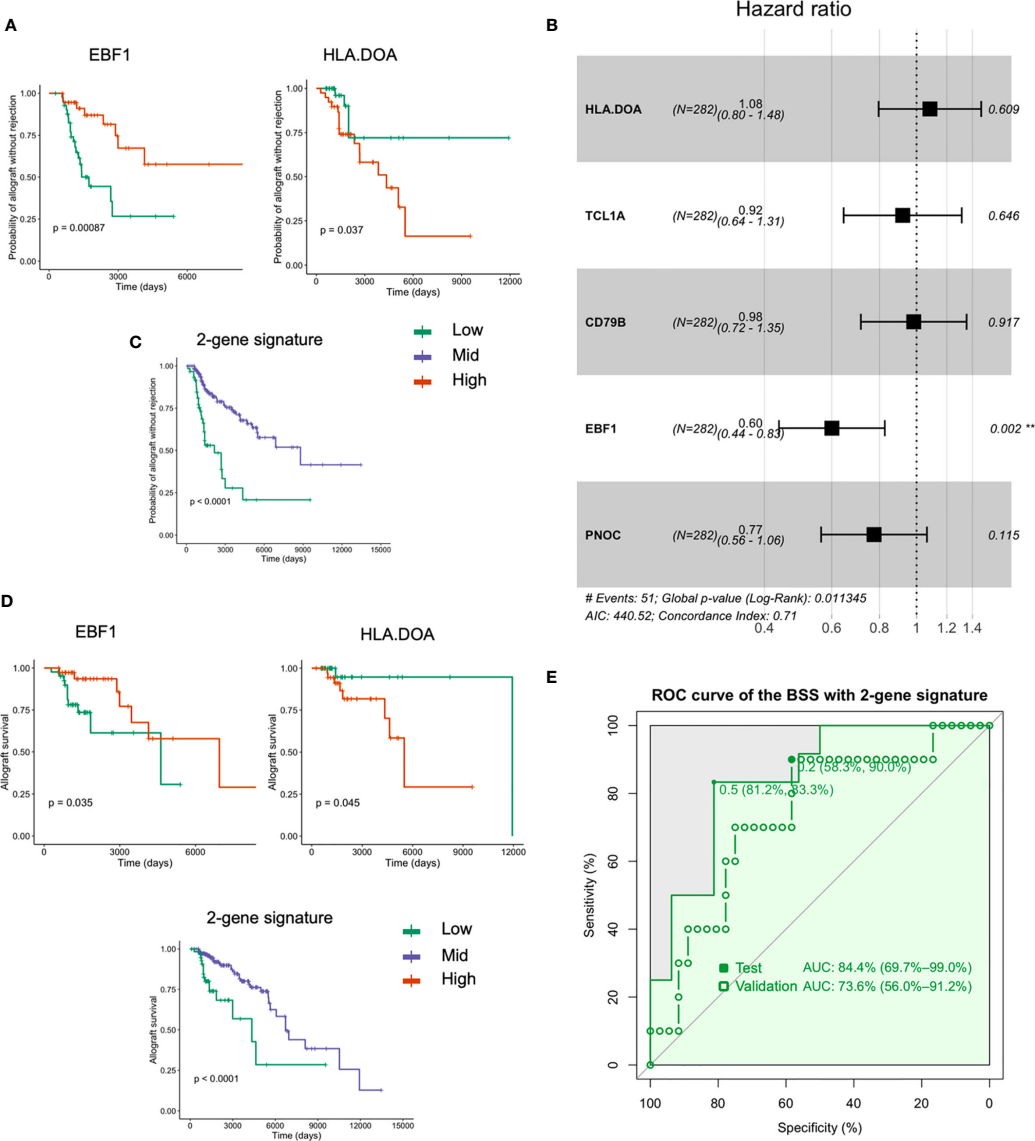
HLA-DOA and EBF1 are associated with graft rejection and allograft survival. **(A)** The survival curves for HLA-DOA and EBF1 are plotted (n = 282). **(B)** EBF1 independently predicted renal graft rejection. **(C)** Patients with a low expression of the 2-gene signature had poor outcomes, while those with higher expression had a longer graft with stable function. **(D)** The rejection curve for HLA-DOA and EBF1 are plotted. The patients with a low expression of the 2-gene signature experienced faster graft loss. **(E)** An AUC of 84.4% could be achieved using EBF1 and HLA-DOA with the BSS model, while an AUC value of 73.6% could be achieved in the validation dataset 2. **P < 0.01.

Using the Z-scale cut-offs obtained in the Cox analysis, subjects were divided into high-, mid-, and low- expression groups as described in the methods. Among the five gene features (HLA-DOA, TCL1A, EBF1, CD79B, and PNOC) used in the BSS model, HLA-DOA and EBF1 were found to be significantly associated with renal allograft rejection (FDR-adjusted *P* < 0.05). The effects of HLA-DOA and EBF1 on the overall survival are plotted in **Figure 4A**. To further investigate whether the genes exert an independent effect on graft rejection and survival, a proportional hazards model was applied. We discovered that EBF1 independently predicted graft rejection (**Figure 4B**). Additionally, patients in the group with a low expression of the 2-gene signature had poor outcomes, while higher expression was associated with a longer surviving graft with stable function (**Figure 4C**). Furthermore, HLA-DOA and EBF1 were included in or selected by all the ML models that were successful in predicting renal allograft loss (**Figure 4D**). An AUC of 84.4% was achieved using the two-gene signature for tolerance prediction (sensitivity=0.83, specificity=0.81) using BSS in the discovery dataset 1, while the AUC was 73.6% in the validation dataset 2 (**Figure 4E**).

## Discussion

Analysis of long-term renal allograft tolerance induction in human subjects remains challenging, largely due to an extremely limited number of successful participants in existing tolerance studies (3). Although allograft tolerance has been studied with ML algorithms using LDA, existing studies have had genome-wide expression data on a relatively small sample size (3, 10), which in turn has curtailed the power of ML to robustly forecast the genomic factors associated with tolerance. To address this challenge, we merged data from three different genome-wide expression studies. The resulting transcriptomics dataset (GSE166865) together with IOT and ITN, being made publicly available with this work, includes 63 total tolerant patients, the largest sample size to date among all human renal allograft tolerance studies for ML exploration.

Different ML methods vary not only in their predictive abilities, but also in terms of the penalty they impose on features that are not as informative in forecasting the output. The latter naturally allows for feature selection in a complex, high-dimensional space, and consequently a comparison of diverse models allows for the selection of optimal biomarkers for clinical application. Using the most statistically significant 31 DEGs between tolerance and chronic rejection (CR), the Brouad group was successfully able to discern tolerance from CR with a specificity of 99% (5, 31). Identifying individuals with allograft tolerance with high confidence is important for clinicians to safely minimize or withdraw immunosuppression without rejection (6). Sagoo et al. had previously identified 10 DEGs among TOL, STA, CR, and HV groups that resulted in a sensitivity of 80.6% and a specificity of 89.0% for their predictive model (7). Using elastic nets, they reported a 9-gene signature that further increased the model sensitivity to 92% and specificity to 88% (10). Similarly, using LDA Newell et al. defined three B cell-related genes (IGKV4-1, IGLL1, and IGKV1D-13) from 249 DEGs, and found that this 3-gene signature resulted in a PPV of 83% and an NPV of 84% (3). Subsequently, they found that IGKV1D-13 showed a consistent increase in patients rendered tolerant *via* chimerism induction, and those individuals maintained minimal immunosuppression akin to spontaneously tolerant patients (32). Building on these efforts, 24 B cell-related genes have been implicated as informative in enhancing the predictive ability of ML models (8). Using logistic regression, three of these transcript signatures (KLF6, BNC2, and CYP1B1) have been found to accurately classify the TOL and STA individuals with a sensitivity of 84.6% and a specificity of 90.2% (33). In the current study, we systematically examined 14 different ML models and found that BSS obtained a sensitivity of 91.7% and a specificity of 93.8%. These predictive statistics were robust across a range of cross-validations, and to our knowledge, outperform existing published ML models that have sought to predict renal allograft tolerance. Notably, different immunosuppression time (156 *vs*. 7 months) between the 2 groups may affect the differential expression of genes. To eliminate the potential influence, we combined the 2 groups to obtain a stably expressed gene signature across the groups. However, further research on immunosuppression time is still needed to examine its importance on DEGs.

Among the BSS selected set of five genes, four B cell-specific genes – HLA-DOA, TCL1A, EBF1, and CD79B – were further confirmed as being predictive of tolerance status, insinuating at the vital role of B cells in promoting and maintaining tolerance. TCL1A, PNOC, and CD79B have been reported as valuable biomarkers of tolerance in prior studies (7, 8, 12). For example, TCL1A expression has been reported to be highest in immature cells, and lower or even absent in mature B cells (34). Herein we found that TCL1A expression was increased in the PBMCs of TOL patients compared to the STA patients. This is in accordance with the literature wherein increased TCL1A expression has been observed in the STA PBMCs, and decreased TCL1A has been reported in both the PBMCs and isolated B cells in acute rejection (AR) (35, 36).

The B cell-specific genes that comprise our model are particularly revealing. For instance, the B-cell-related biomarker of tolerance EBF1 is upregulated in the tolerant patients when compared to the STA patients. EBF1 is crucial for B lineage commitment (37). Choi et al. performed gene expression analysis on subjects with AR and TOL, and found that both EBF1 and TCL1A were upregulated in the tolerant patients, highlighting their role in tolerance induction (38). Herein we found that EBF1 expression was upregulated in tolerant patients in comparison with STA patients, and EBF1 could also predict the chronic graft rejection and graft loss. This suggests a possible role for EBF1 in B cell-mediated tolerance and renal graft survival. Similarly, HLA-DOA, together with HLA-DOB, encodes HLA-DO (39), and inhibits B cell-mediated antigen presentation (41). Downregulation of HLA-DOA in children after liver transplantation enhances antigen-presentation by B cells (40, 41). Yet, whether and how HLA-DOA might affect allograft tolerance has remained unclear. Here we found that HLA-DOA is an important predictive feature in the BSS model, and tolerant patients had a significantly higher HLA-DOA expression in comparison to STA. Survival analysis using the two-gene signature (EBF1 and HLA-DOA) further substantiates an important role for EBF1 and HLA-DOA, and points to novel hypotheses that can be readily tested experimentally.

## Conclusions

We compared 14 different machine learning models for renal allograft tolerance prediction using genomic features from PBMC microarray data, and found that Best Subset Selection (BSS) was the most robust method for tolerance prediction with both specificity and sensitivity > 90%. We also identified a novel feature set consisting of five genes, four of which were B cell-related, that consistently predicted tolerance and resulted in better ML performance than other existing models. Our findings collectively provide clinically actionable insights that can guide practitioners on novel biomarkers associated with tolerance, and consequently identify patients for whom immunosuppression withdrawal would have a relatively low risk of acute or choric rejection.

## Data Availability Statement

The datasets presented in this study can be found in online repositories. The names of the repository/repositories and accession number(s) can be found below: https://www.ncbi.nlm.nih.gov/, GSE166865.

## Author Contributions

QF, SD, and JM: study design. QF: sample and data acquisition. QF: statistical analysis. QF: drafting of the manuscript. DA, KD, RM, HY, and JM: revising of the manuscript. LW, QR, and JM: obtained funding. All authors contributed to the article and approved the submitted version.

## Funding

This work was supported by grants from the Department of Science and Technology of Sichuan Province (No. 30504010361), Science Fund for Distinguished Young Scholars of Sichuan Province (No. 2020JDJQ0066), and NIH/NIAID, No. 2R01AI057851-12A1 (JFM).

## Conflict of Interest

The authors declare that the research was conducted in the absence of any commercial or financial relationships that could be construed as a potential conflict of interest.

## Abbreviations

AR, acute rejection; AUC, area under the curve; BIC, Bayesian Information Criterion; BSS, best subset selection; CR, chronic rejection; DEGs, Differentially expressed genes; E-Net, elastic network; GEO, Gene Expression Omnibus; GO, Gene Ontology; HV, healthy volunteers; KKNN, weighted k-Nearest Neighbor; KNN, k-Nearest Neighbor; LDA, Linear Discriminant Analysis; LOOVE, leave-one-out cross-validation; MARS, Multivariate Adaptive Regression Splines; ML, machine learning; NPV, negative predictive value; PBL, peripheral blood lymphocyte; PBMCs, peripheral blood mononuclear cells; PCA, principal component analysis; PCs, Principal components; PPV, positive predictive value; QDA, Quadratic discriminant analysis; RBF, radial basis function; SI, standard immunotherapy; SVM, support vector machine; RBF, radial basis function; XGBoost, eXtreme Gradient Boosting.

## References

[1] Pascual M Theruvath T Kawai T Tolkoff-Rubin N Cosimi AB. Strategies to Improve Long-Term Outcomes After Renal Transplantation. N Engl J Med (2002) 346(8):580–90. 10.1056/NEJMra011295 11856798

[2] Wheeler DC Steiger J. Evolution and Etiology of Cardiovascular Diseases in Renal Transplant Recipients. Transplantation (2000) 70(11 Suppl):SS41–5. 11152230

[3] Newell KA Asare A Kirk AD Gisler TD Bourcier K Suthanthiran M. Identification of a B Cell Signature Associated With Renal Transplant Tolerance in Humans. J Clin Invest (2010) 120(6):1836–47. 10.1172/JCI39933 PMC2877933 20501946

[4] Lechler RI Garden OA Turka LA. The Complementary Roles of Deletion and Regulation in Transplantation Tolerance. Nat Rev Immunol (2003) 3(2):147–58. 10.1038/nri1002 12563298

[5] Brouard S Mansfield E Braud C Li L Giral M Hsieh SC. Identification of a Peripheral Blood Transcriptional Biomarker Panel Associated With Operational Renal Allograft Tolerance. Proc Natl Acad Sci USA (2007) 104(39):15448–53. 10.1073/pnas.0705834104 PMC2000539 17873064

[6] Braud C Baeten D Giral M Pallier A Ashton-Chess J Braudeau C. Immunosuppressive Drug-Free Operational Immune Tolerance in Human Kidney Transplant Recipients: Part I. Blood Gene Expression Statistical Analysis. J Cell Biochem (2008) 103(6):1681–92. 10.1002/jcb.21574 17910029

[7] Sagoo P Perucha E Sawitzki B Tomiuk S Stephens DA Miqueu P. Development of a Cross-Platform Biomarker Signature to Detect Renal Transplant Tolerance in Humans. J Clin Invest (2010) 120(6):1848–61. 10.1172/JCI39922 PMC2877932 20501943

[8] Lozano JJ Pallier A Martinez-Llordella M Danger R Lopez M Giral M. Comparison of Transcriptional and Blood Cell-Phenotypic Markers Between Operationally Tolerant Liver and Kidney Recipients. Am J Transplant (2011) 11(9):1916–26. 10.1111/j.1600-6143.2011.03638.x 21827613

[9] Hricik DE Formica RN Nickerson P Rush D Fairchild RL Poggio ED. Adverse Outcomes of Tacrolimus Withdrawal in Immune-Quiescent Kidney Transplant Recipients. J Am Soc Nephrol (2015) 26(12):3114–22. 10.1681/ASN.2014121234 PMC4657844 25925687

[10] Rebollo-Mesa I Nova-Lamperti E Mobillo P Runglall M Christakoudi S Norris S. Biomarkers of Tolerance in Kidney Transplantation: are We Predicting Tolerance or Response to Immunosuppressive Treatment? Am J Transplant (2016) 16(12):3443–57. 10.1111/ajt.13932 PMC5132071 27328267

[11] Roussey-Kesler G Giral M Moreau A Subra JF Legendre C Noel C. Clinical Operational Tolerance After Kidney Transplantation. Am J Transplant (2006) 6(4):736–46. 10.1111/j.1600-6143.2006.01280.x 16539630

[12] Baron D Ramstein G Chesneau M Echasseriau Y Pallier A Paul C. A Common Gene Signature Across Multiple Studies Relate Biomarkers and Functional Regulation in Tolerance to Renal Allograft. Kidney Int (2015) 87(5):984–95. 10.1038/ki.2014.395 PMC4424816 25629549

[13] Shabalin AA Tjelmeland H Fan C Perou CM Nobel AB. Merging Two Gene-Expression Studies *via* Cross-Platform Normalization. Bioinformatics (2008) 24(9):1154–60. 10.1093/bioinformatics/btn083 18325927

[14] Angel PW Rajab N Deng Y Pacheco CM Chen T Le Cao KA. A Simple, Scalable Approach to Building a Cross-Platform Transcriptome Atlas. PloS Comput Biol (2020) 16(9):e1008219. 10.1371/journal.pcbi.1008219 32986694 PMC7544119

[15] Spann A Yasodhara A Kang J Watt K Wang B Goldenberg A. Applying Machine Learning in Liver Disease and Transplantation: a Comprehensive Review. Hepatology (2020) 71(3):1093–105. 10.1002/hep.31103 31907954

[16] Wei R Wang J Wang X Xie G Wang Y Zhang H. Clinical Prediction of HBV and HCV Related Hepatic Fibrosis Using Machine Learning. EBioMedicine (2018) 35:124–32. 10.1016/j.ebiom.2018.07.041 PMC6154783 30100397

[17] Berglund E Lytsy P Westerling R. Adherence to and Beliefs in Lipid-Lowering Medical Treatments: a Structural Equation Modeling Approach Including the Necessity-Concern Framework. Patient Educ Couns (2013) 91(1):105–12. 10.1016/j.pec.2012.11.001 23218590

[18] Xiao J Ding R Xu X Guan H Feng X Sun T. Comparison and Development of Machine Learning Tools in the Prediction of Chronic Kidney Disease Progression. J Transl Med (2019) 17(1):119. 10.1186/s12967-019-1860-0 30971285 PMC6458616

[19] Wu Z Irizarry RA Gentleman R Martinez-Murillo F Spencer F. A Model-Based Background Adjustment for Oligonucleotide Expression Arrays. J Am Stat Assoc (2004) 99(468):909–17. 10.1198/016214504000000683

[20] Hastie T Tibshirani R Narasimhan B Chu G. Impute: Imputation for Microarray Data. R package version 1.66.0. (2016) 17:520–5. 10.18129/B9.bioc.impute

[21] Johnson WE Li C Rabinovic A. Adjusting Batch Effects in Microarray Expression Data Using Empirical Bayes Methods. Biostatistics (2007) 8(1):118–27. 10.1093/biostatistics/kxj037 16632515

[22] Ritchie ME Phipson B Wu D Hu Y Law CW Shi W. Limma Powers Differential Expression Analyses for RNA-Sequencing and Microarray Studies. Nucleic Acids Res (2015) 43(7):e47. 10.1093/nar/gkv007 25605792 PMC4402510

[23] Venables WN Ripley BD. Linear Statistical Models. In: Modern Applied Statistics with S. Statistics and Computing. New York, NY: Springer (2002). 10.1007/978-0-387-21706-2_6

[24] Friedman JH Silverman BW. Flexible Parsimonious Smoothing and Additive Modeling. Technometrics (1989) 31(1):3–21. 10.2307/1270359

[25] Tibshirani R Bien J Friedman J Hastie T Simon N Taylor J. Strong Rules for Discarding Predictors in Lasso-Type Problems. J R Stat Soc Ser B Stat Methodol (2012) 74(2):245–66. 10.1111/j.1467-9868.2011.01004.x PMC4262615 25506256

[26] Breiman L. Random Forests. Mach Learn (2001) 45(1):5–32. 10.1023/A:1010933404324

[27] Chen T Guestrin C. XGBoost: a Scalable Tree Boosting System. In: KDD ‘16: Proceedings of the 22nd ACM SIGKDD International Conference on Knowledge Discovery and Data Mining (2016). pp. 785–94. 10.1145/2939672.2939785

[28] William R. Psych: Procedures for Psychological, Psychometric, and Personality Research. R package version 1.9.12. Evanstone, Illinois: Northwestern University (2019).

[29] Agarwal D Zhang NR. Semblance: an Empirical Similarity Kernel on Probability Spaces. Sci Adv (2019) 5(12):eaau9630. 10.1126/sciadv.aau9630 31840051 PMC6892634

[30] Einecke G Reeve J Sis B Mengel M Hidalgo L Famulski KS. A Molecular Classifier for Predicting Future Graft Loss in Late Kidney Transplant Biopsies. J Clin Invest (2010) 120(6):1862–72. 10.1172/JCI41789 PMC2877953 20501945

[31] Tibshirani R Hastie T Narasimhan B Chu G. Diagnosis of Multiple Cancer Types by Shrunken Centroids of Gene Expression. Proc Natl Acad Sci USA (2002) 99(10):6567–72. 10.1073/pnas.082099299 PMC124443 12011421

[32] Newell KA Asare A Sanz I Wei C Rosenberg A Gao Z. Longitudinal Studies of a B Cell-Derived Signature of Tolerance in Renal Transplant Recipients. Am J Transplant (2015) 15(11):2908–20. 10.1111/ajt.13480 PMC4725587 26461968

[33] Roedder S Li L Alonso MN Hsieh SC Vu MT Dai H. A Three-Gene Assay for Monitoring Immune Quiescence in Kidney Transplantation. J Am Soc Nephrol (2015) 26(8):2042–53. 10.1681/ASN.2013111239 PMC4520154 25429124

[34] Kuraishy AI French SW Sherman M Herling M Jones D Wall R. TORC2 Regulates Germinal Center Repression of the TCL1 Oncoprotein to Promote B Cell Development and Inhibit Transformation. Proc Natl Acad Sci U.S.A. (2007) 104(24):10175–80. 10.1073/pnas.0704170104 PMC1891214 17548807

[35] Heidt S Vergunst M Anholts JD Reinders ME de Fijter JW Eikmans M. B Cell Markers of Operational Tolerance can Discriminate Acute Kidney Allograft Rejection From Stable Graft Function. Transplantation (2015) 99(5):1058–64. 10.1097/TP.0000000000000465 25340606

[36] Viklicky O Krystufkova E Brabcova I Sekerkova A Wohlfahrt P Hribova P. B-Cell-Related Biomarkers of Tolerance are Up-Regulated in Rejection-Free Kidney Transplant Recipients. Transplantation (2013) 95(1):148–54. 10.1097/TP.0b013e3182789a24 23222918

[37] Zandi S Mansson R Tsapogas P Zetterblad J Bryder D Sigvardsson M. EBF1 is Essential for B-Lineage Priming and Establishment of a Transcription Factor Network in Common Lymphoid Progenitors. J Immunol (2008) 181(5):3364–72. 10.4049/jimmunol.181.5.3364 18714008

[38] Choi JW Kim YH Oh JW. Comparative Analyses of Signature Genes in Acute Rejection and Operational Tolerance. Immune Netw (2017) 17(4):237–49. 10.4110/in.2017.17.4.237 PMC5577301 28860953

[39] Nagarajan UM Lochamy J Chen X Beresford GW Nilsen R Jensen PE. Class II Transactivator is Required for Maximal Expression of HLA-DOB in B Cells. J Immunol (2002) 168(4):1780–6. 10.4049/jimmunol.168.4.1780 11823510

[40] Ningappa M Ashokkumar C Higgs BW Sun Q Jaffe R Mazariegos G. Enhanced B Cell Alloantigen Presentation and its Epigenetic Dysregulation in Liver Transplant Rejection. Am J Transplant (2016) 16(2):497–508. 10.1111/ajt.13509 26663361 PMC5082419

[41] Sindhi R Higgs BW Weeks DE Ashokkumar C Jaffe R Kim C. Genetic Variants in Major Histocompatibility Complex-Linked Genes Associate With Pediatric Liver Transplant Rejection. Gastroenterology (2008) 135(3):830–9, 839.e1–10. 10.1053/j.gastro.2008.05.080 18639552 PMC2956436

