# Supplementary material for: An Unbiased Machine Learning Exploration Reveals Gene Sets Predictive of Allograft Tolerance After Kidney Transplantation
Source: Front Immunol. 2021 Jul 8;12:695806. doi: 10.3389/fimmu.2021.695806 (PMC8297499; doi:10.3389/fimmu.2021.695806)

**Supplementary Figures and Tables**

**Supplementary Figures**

**
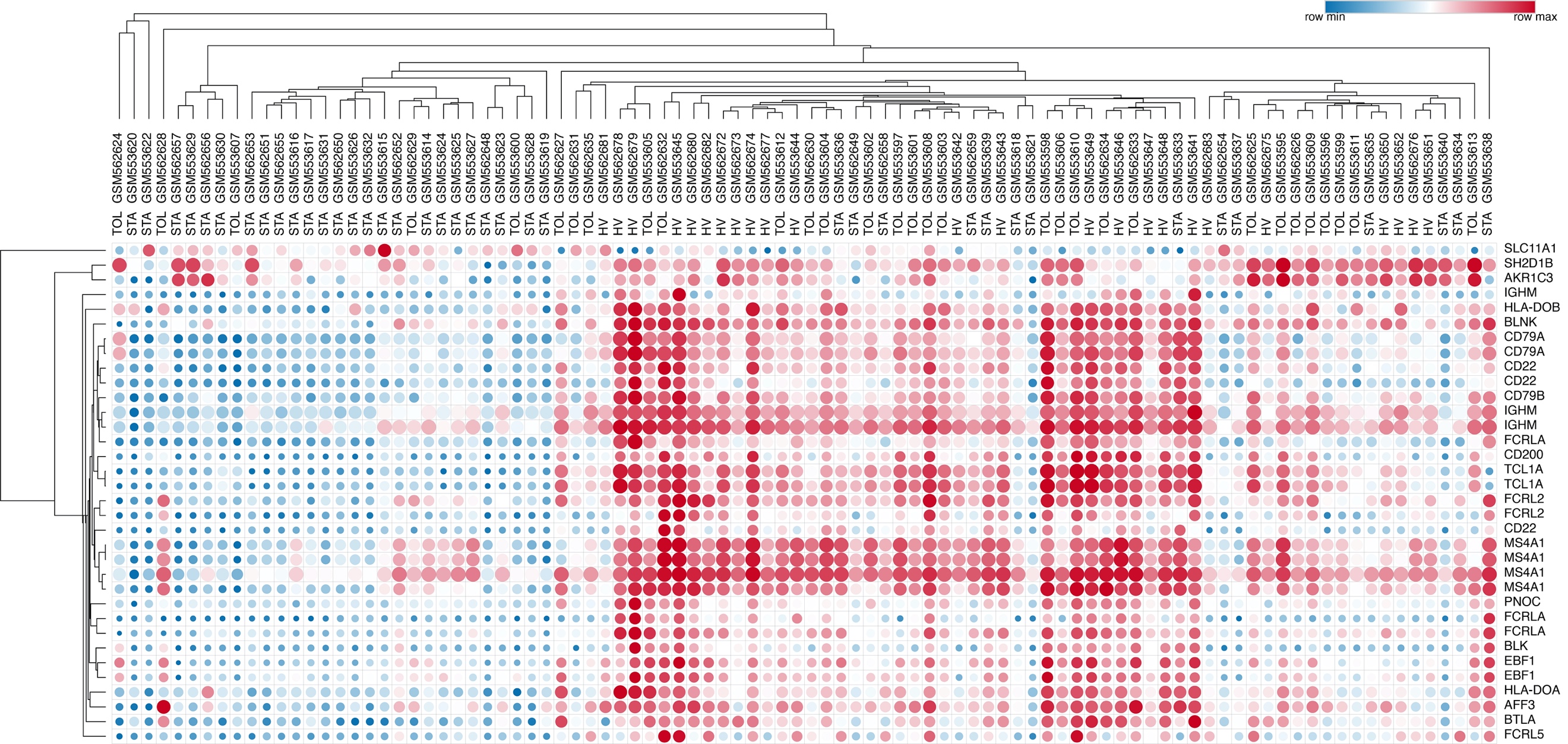
**

**Figure S1. The heatmap of the co-DEGs between the dataset 1 and dataset 2 (ITN and IOT).**

**
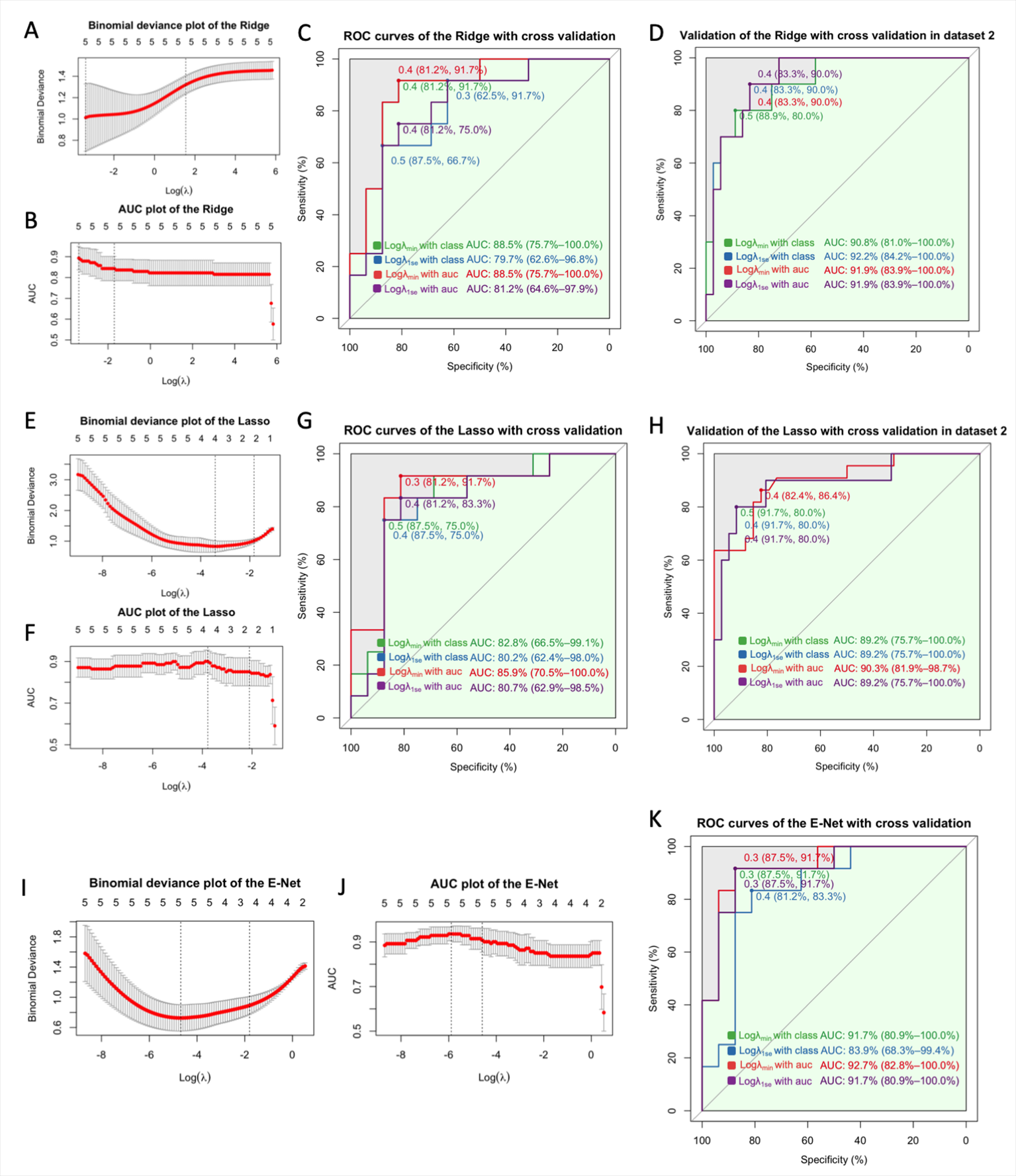
**

**Figure S2. Cross-validation of the Ridge, Lasso, and E-Net models.**

The binomial deviance plot (A), the AUC plot (B), and the cross-validated ROC curves (C) after k-fold cross-validation (k=5) of the Ridge regression model are shown. The ROC curves of the Ridge (D) validated in dataset 2 are also demonstrated. The binomial deviance plot (E), AUC plot (F), and the cross-validated ROC curves (G) of the Lasso model, as well as the ROC curves of the Lasso (H) validated in dataset 2 are shown. The binomial deviance plot (I), AUC plot (I), and ROC curves with cross-validation (J) of the E-Net model are depicted here. k-fold cross-validation with k=5 was used with λ1se or λmin when the type measure is “class” or “auc”. The left dotted line represents the minimum binomial deviance when using Log λmin and the right dotted line represents the binomial deviance one standard error away from the minimum when using Log λ1se.

**
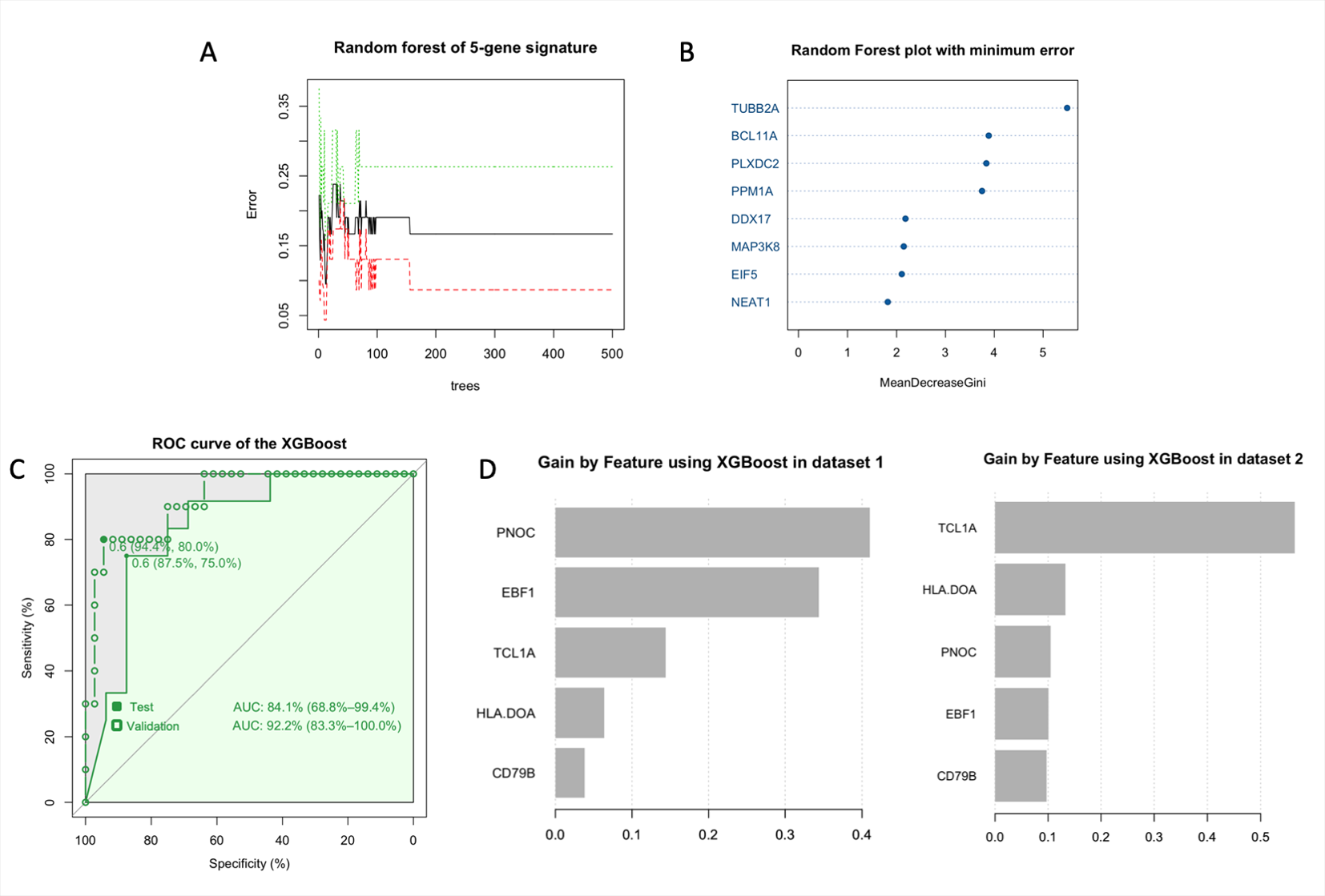
**

**Figure S3. Parameters of Random forest and Prediction assessment of the XGBoost model.**

(A) The random forest model obtained a minimum error and the highest accuracy rate of 71.43% when tree number was 12. (B) The gene features are ordered based on the MeanDecreaseGini value. (C) XGBoost has an AUC value of 84.1% in dataset 1 and 92.2% in dataset 2. (D) The gain by additional features in the XGBoost model in datasets 1 and 2 is shown.

**
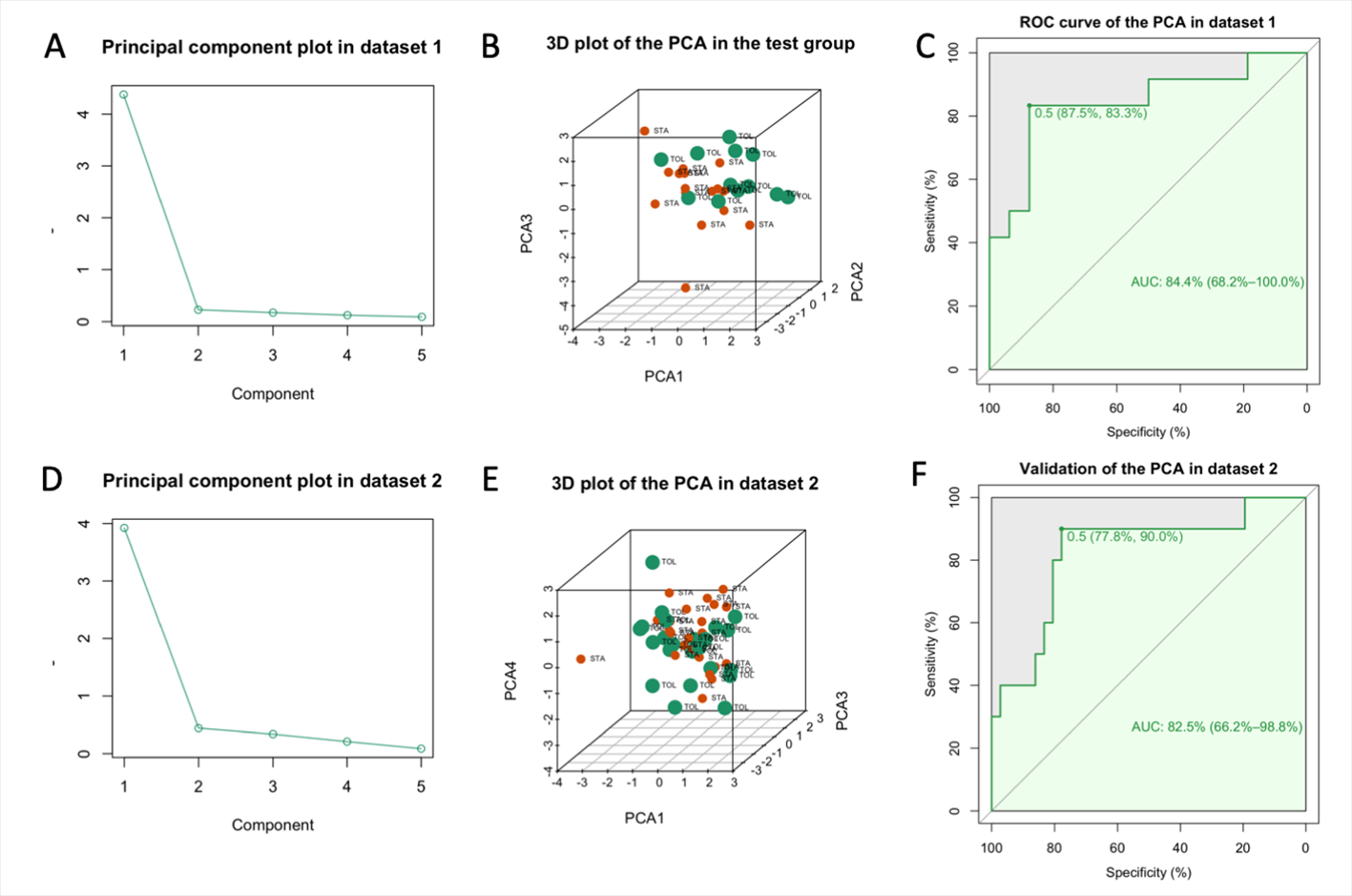
**

**Figure 6. Prediction assessment and validation of the PCA model.**

(A) The first three principal components explain ~80% variance. (B) 3D PCA plot of the prediction using the top three PCs. These top three PCs separated TOL from STA for the majority of patients in the test group. (C) An AUC value of 84.4% with a sensitivity of 83.3% and a specificity of 84.4% were achieved using the top three PCs. (D) PCA plot of the tolerance prediction using the top three PCs in dataset 2. (F) A similarly high AUC value with a sensitivity of 90% and a specificity of 77.8% were achieved using the top three PCs in dataset 2.

**Supplementary Tables**

**Supplementary Table S1. Demographic characteristics of patient groups**


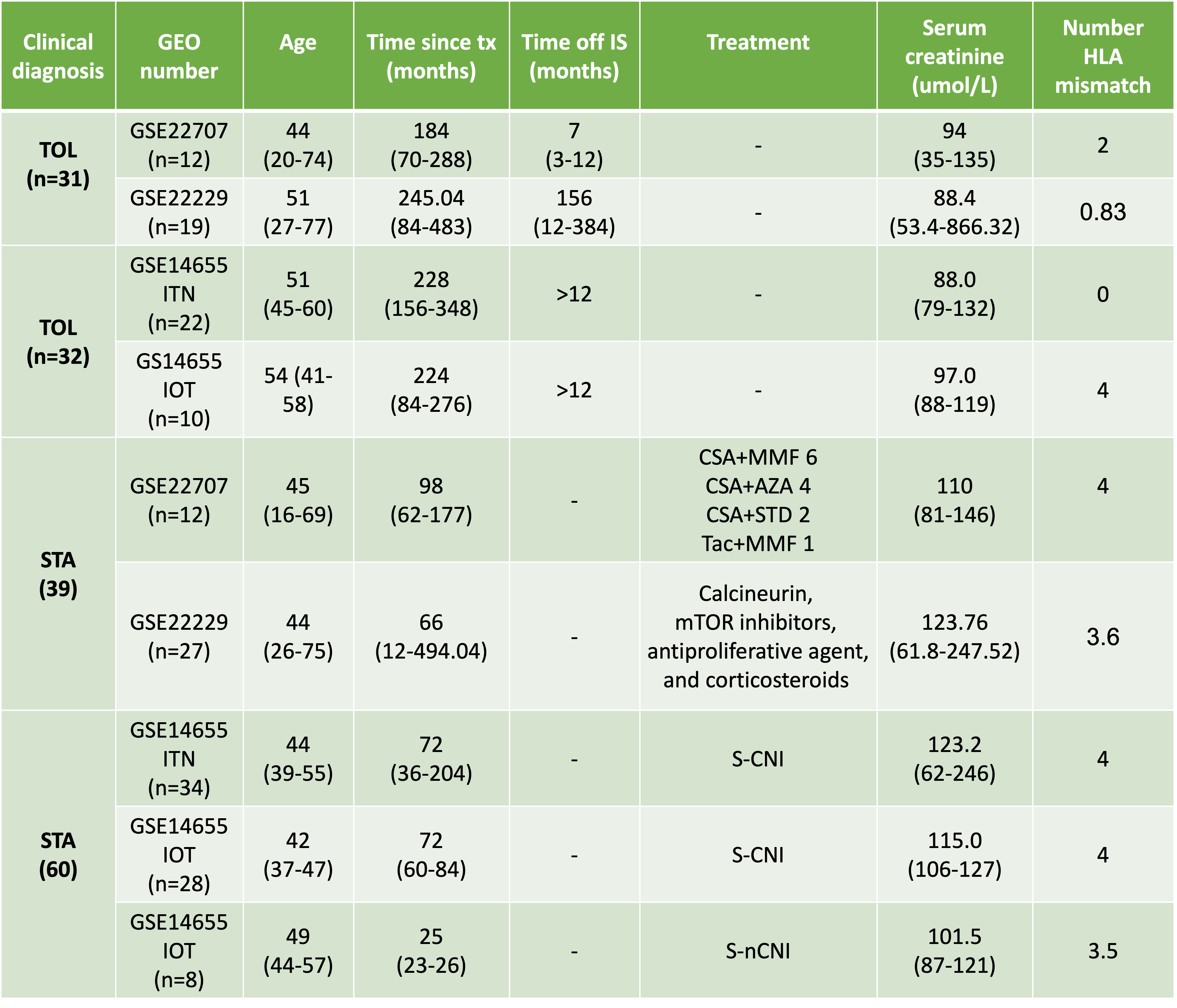


**Supplementary Table S1 Genes selected in the BSS model**

**Supplementary Table S2. Co-Genes and signature selected in the BSS model (Top 5)**


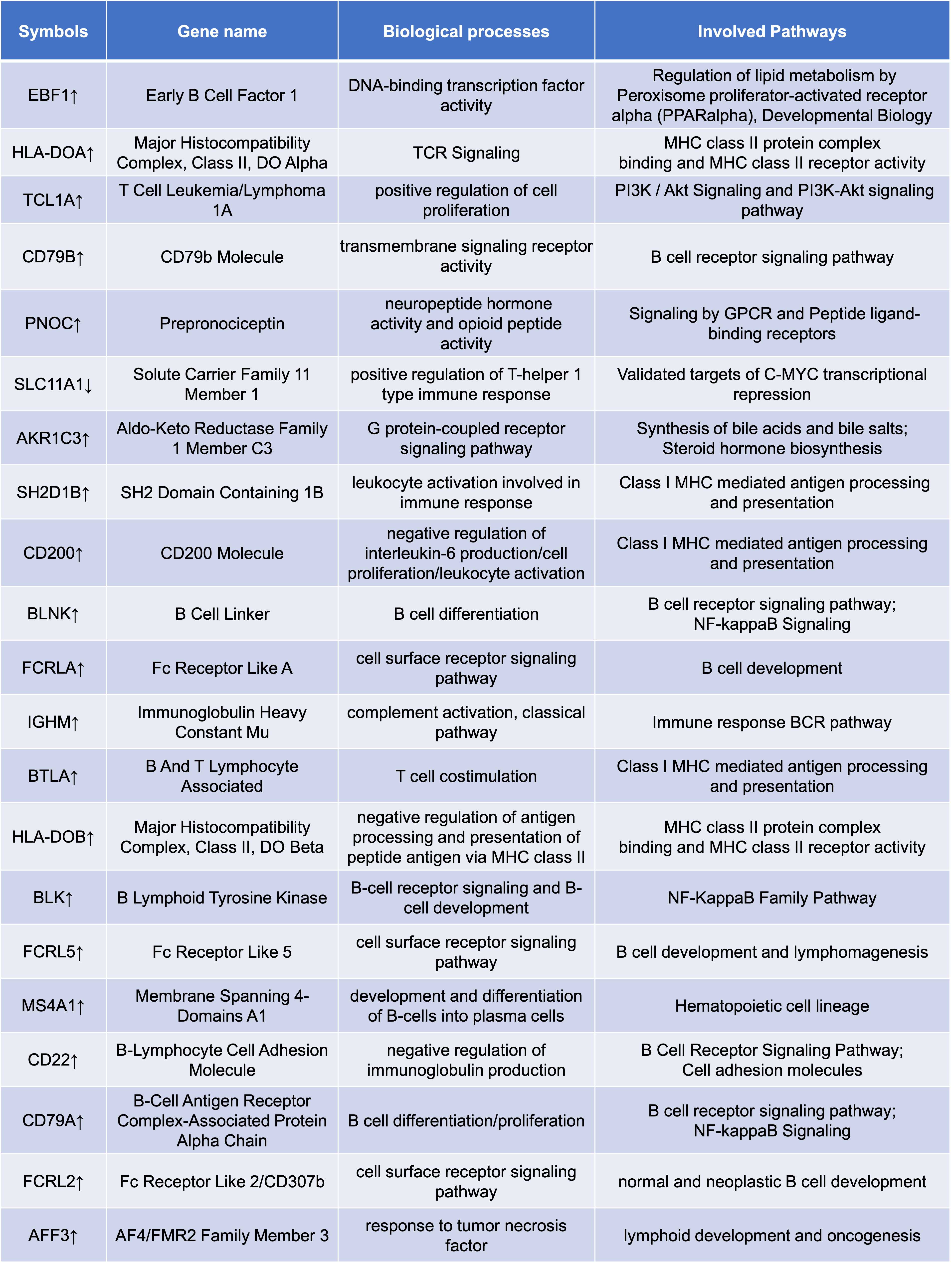

Supplement: Supplementary file 1 [file DataSheet_1.docx]
